# Characterization of Aspartate Kinase from *Corynebacterium* *pekinense* and the Critical Site of Arg169

**DOI:** 10.3390/ijms161226098

**Published:** 2015-11-27

**Authors:** Weihong Min, Huiying Li, Hongmei Li, Chunlei Liu, Jingsheng Liu

**Affiliations:** 1 College of Food Science and Engineering, Jilin Agricultural University, Changchun 130118, China; lihuiying@jlau.edu.cn (H.L.); lihongmei@jlau.edu.cn (H.L.); liuchunlei0709@jlau.edu.cn (C.L.); liujs2000@jlau.edu.cn (J.L.); 2 National Engineering Laboratory of Wheat and Corn Deep Processing, Changchun 130118, China

**Keywords:** *Corynebacterium pekinense*, aspartate kinase, characterization, molecular docking

## Abstract

Aspartate kinase (AK) is the key enzyme in the biosynthesis of aspartate-derived amino acids. Recombinant AK was efficiently purified and systematically characterized through analysis under optimal conditions combined with steady-state kinetics study. Homogeneous AK was predicted as a decamer with a molecular weight of ~48 kDa and a half-life of 4.5 h. The enzymatic activity was enhanced by ethanol and Ni^2+^. Moreover, steady-state kinetic study confirmed that AK is an allosteric enzyme, and its activity was inhibited by allosteric inhibitors, such as Lys, Met, and Thr. Theoretical results indicated the binding mode of AK and showed that Arg169 is an important residue in substrate binding, catalytic domain, and inhibitor binding. The values of the kinetic parameter *V*_max_ of R169 mutants, namely, R169Y, R169P, R169D, and R169H AK, with l-aspartate as the substrate, were 4.71-, 2.25-, 2.57-, and 2.13-fold higher, respectively, than that of the wild-type AK. Furthermore, experimental and theoretical data showed that Arg169 formed a hydrogen bond with Glu92, which functions as the entrance gate. This study provides a basis to develop new enzymes and elucidate the corresponding amino acid production.

## 1. Introduction

Aspartate kinase (AK) is the first identified important enzyme in the biosynthesis of several amino acids, such as Lys, Thr, and Met, which are essential to mammals. AK is a multimer and an allosteric enzyme that catalyzes the phosphorylation of aspartate to form aspartyl-P [1,2,3]. Allosteric regulation (feedback inhibition) of proteins controls the synthesis pathway of this enzyme [4]. AK is classified based on subunit organization as a homo-oligomer or heterotetramer. AK can also be classified into three types, namely AK-III, AK-II, and AK, depending on the source organism [5,6]. AK-III from *Escherichia coli*, AK-I from *Arabidopsis thaliana*, and AK from *Methanocaldococcus jannaschii* are homo-oligomers; furthermore, AK from *Thermus thermophilus*, AK-II from *Bacillus subtilis*, and AK from *Corynebacterium glutamicum* are heterotetramers [7,8,9].

**Figure 1:**
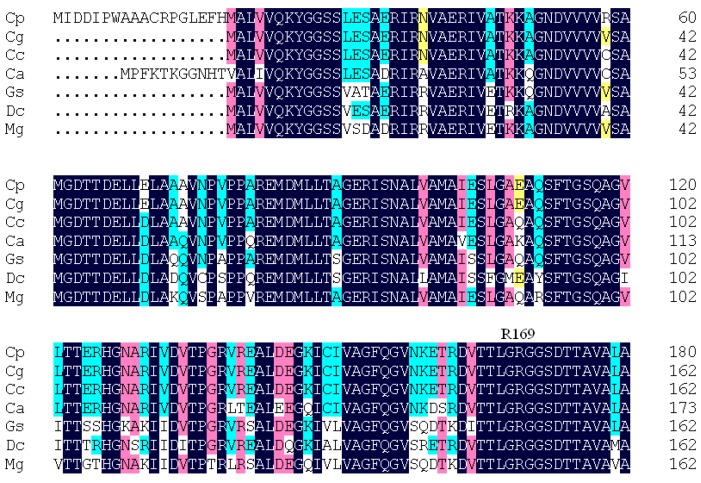
Multiple sequence alignment of aspartate kinase (AK) with other members. CpAK from *C. pekinense*; CgAK from *C. glutamicum*, 99%; CcAK from *C. callunae*, 94%; CaAK from *C. accolens*, 87%; GsAK from *Gordonia sputi*, 77%; DcAK from *Dietzia cinnamea*, 76%; and MgAK from *Mycobacterium gilvum*, 74%. Percentages represent the degree of amino acid sequence identity. In ascending order, sequence similarity is highlighted in yellow, sky blue, pink, and blue.

AK from *Corynebacterium pekinense* (CpAK) shares 98.5% sequence identity with AK from *C. glutamicum* (AK-II), with an α_2_β_2_-type structure containing two α and β subunits [4,10,11] (Figure 1). Each αβ dimer contains two lysine binding sites [12], in which one site is exclusively found in the dimer with A and B chains [13,14,15] located at the interface between α and β subunits. The presence of this exclusive site indicates that the lysine-binding site in the regulatory region of CgAK performs a vital function in AK allosteric inhibition [16,17].

In this study, we describe CpAK, a class II AK with allosteric effectors, namely Lys and Thr, and determine the regulatory functions of this enzyme. By combining experimental and theoretical data, we reported an important residue, namely Arg169. This residue is involved in enzyme catalysis and forms a hydrogen bond with Glu92, which functions as the entrance gate of AK. This study provides a basis for the design of an allosteric control system for aspartate bioproduction.

## 2. Results and Discussion

### 2.1. Structural Differences between the Active and Inactive Forms of Aspartate Kinase

**Figure 2:**
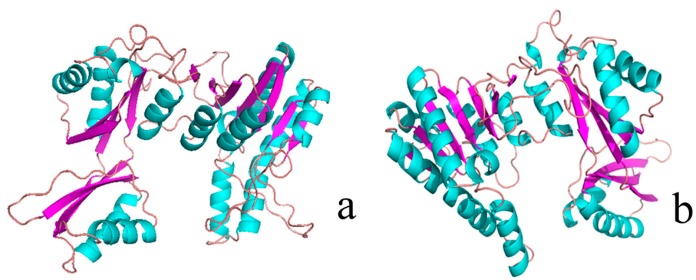

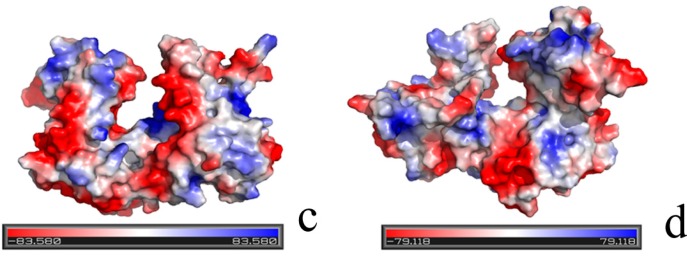
Three-dimensional (3D) structure and protein contact potential of AK (active form: PDB ID 3AB2; inactive form: PDB ID 3AAW). (**a**) 3D structure of the inactive form of AK; (**b**) 3D structure of the active form of AK; color purple represents for β-sheet, and color green represents for α-heliex; (**c**) protein contact potential of the inactive form of AK; and (**d**) protein contact potential of the active form of AK. Red represents negative charge, and blue represents positive charge.

**Figure 3:**
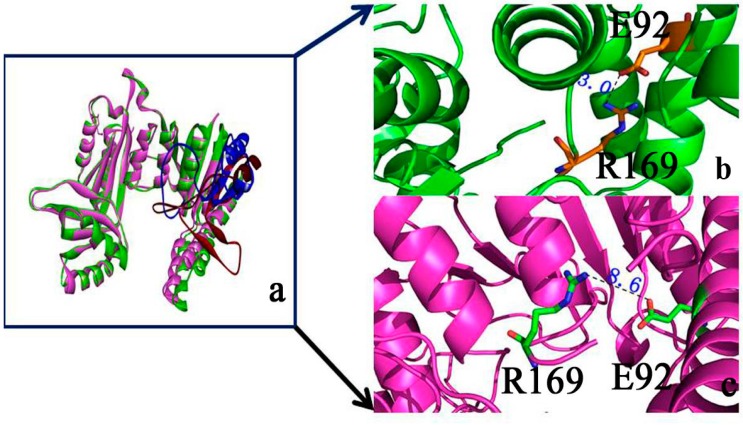
Structure of active/inactive forms of AK. (**a**) Superimposed structures of the active (**purple**) and inactive forms (**green**); color blue represents for open state and color red represents for close state; (**b**) E92 formed a salt bridge with R169 in the inactive form; (**c**) E92 did not form a salt bridge with R169 in the active form.

We constructed AK models by using two crystal structures of AKs (active form: PDB code 3AB2; inactive form: PDB code 3AAW) through Swiss modeler online (sequence identity = 98.5%) [11]. Figure 2a,b show that the two structures were highly similar to each other, but Figure 2c,d indicate that their protein contact potential differed; that is, the active form of AK presented a more compact 3D structure than the inactive form. Figure 3a shows the superimposed structures of the active (purple) and inactive (green) forms. Residues 131 to 171 differed between the two forms. Sequence alignment results showed that Asp92 functions as a catalytic residue [10]. As shown in Figure 3b, Glu92 formed a salt bridge with Lys169 in the inactive form, but no salt bridge was found in the active form (Figure 3c). This structural difference may result in the fluctuation in the catalytic efficiency of AK.

### 2.2. Binding Position of AK

The structure of the substrate aspartic acid was obtained using Gaussian View 4.0 [12] and then optimized with Gaussian 09 software through the B3LYP methods at 6-311G* setting [13]. Aspartic acid was then docked to AK by using Auto Dock Vina.

**Figure 4:**
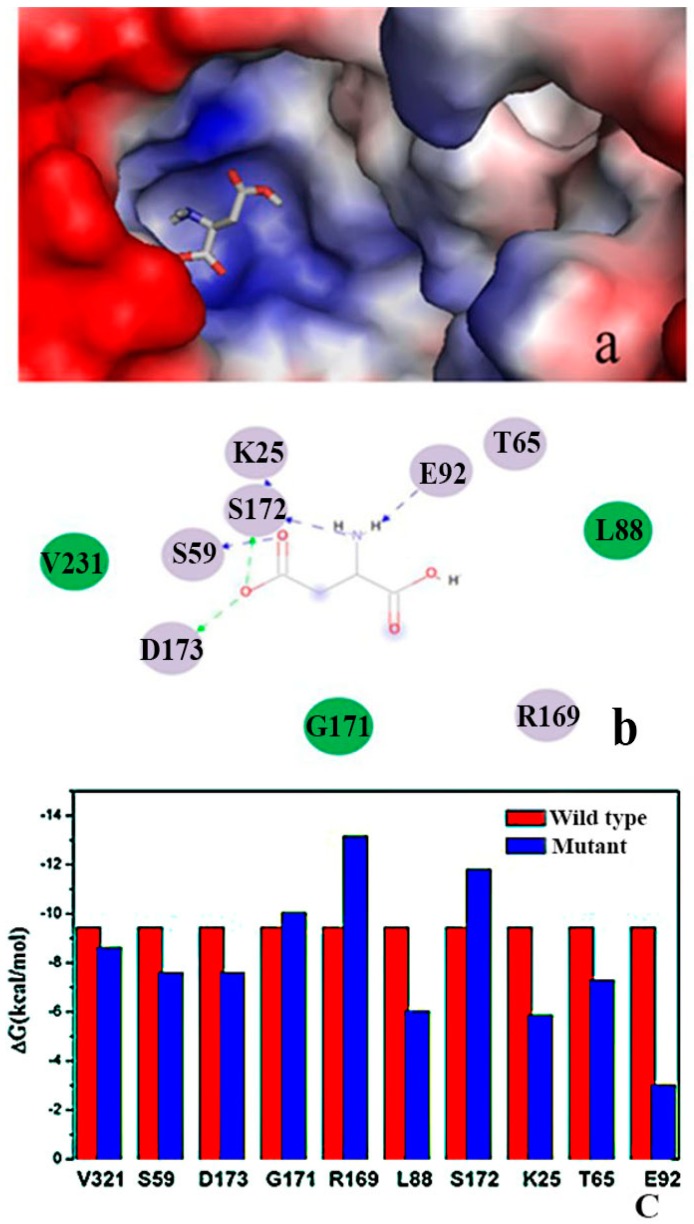
Substrate binding site of AK. (**a**) Aspartic acid located in the active groove. Red represents negative charge, and blue represents positive charge; (**b**) Important residues around aspartic acid binding; color purple represents for electrostatic contacts and color green represents for van der waals contacts; (**c**) Computational alanine mutants (kcal·mol^−1^) calculated for aspartic acid-AK.

Figure 4a shows the substrate docked in the AK structure, with aspartic acid in the groove. Aspartic acid possessed a negative charge and was located in the groove with a positive charge, thereby facilitating charge transfer.

Four hydrogen bonds were present between Asp and AK (Figure 4b). S172 formed two hydrogen bonds with aspartic acid (1.97 and 1.70 Å), whereas D173 formed a hydrogen bond with the O atom with aspartic acid. Another hydrogen bond (2.26 Å) was formed between aspartic acid and K25. Moreover, E92 and S59 formed a hydrogen bond with aspartic acid. Figure 4b shows that V231, L88, and G171 formed electronic contact with aspartic acid, whereas D173, S59, S72, K25, E92, T65, and R169 formed van der Waals interactions with aspartic acid. Therefore, the binding pocket between aspartic acid and AK may contain V231, L88, G171, D173, S59, S72, K25, E92, T65, and R169, with D92 as the catalytic site-binding residue, which is in agreement with the experimental data [10].

The 11 selected residues of the active binding site were used for computational alanine scanning to explore the function of each residue on substrate binding. Figure 4c shows the mutated residues. The R169A mutation decreased the binding affinity of the substrate by 3.72 kcal·mol^−1^. The other main binding components included residues S172 and G171, which present minimal effects when mutated to alanine. Therefore, R169, S172, and G171 may be the key residues in the substrate binding site. We selected R169 for further study.

### 2.3. Expression and Purification of Wild Type and Mutant AK

**Figure 5:**
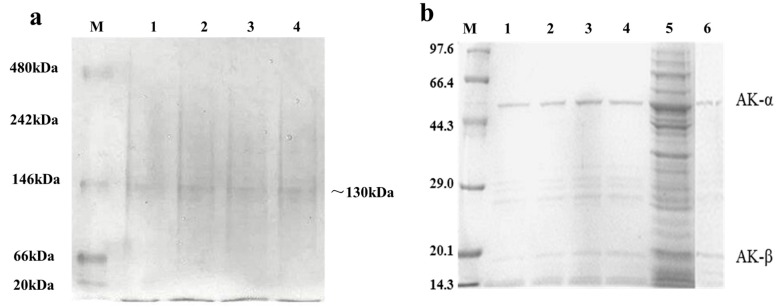
Native polyacrylamide gel electrophoresis (PAGE) and sodium dodecyl sulfate polyacrylamide gel electrophoresis (SDS-PAGE) of the recombinant AK and its mutants. (**a**) Native PAGE of the recombinant AK and the mutants. M: molecular weight marker; lane 1: purified recombinant R169Y; lane 2: purified recombinant R169P; lane 3: purified recombinant R169D; lane 4: purified recombinant R169H; and (**b**) SDS-PAGE of the recombinant AK and the mutants. M: high-molecular weight protein marker; lane 1: purified recombinant R169Y; lane 2: purified recombinant R169P; lane 3: purified recombinant R169D; lane 4: purified recombinant R169H; lane 5: supernatant of induced sample; and lane 6: Western blot of the purified AK.

High-activity mutants, namely, R169Y, R169P, R169D, and R169H, were obtained by high-throughput screening. After native polyacrylamide gel electrophoresis (PAGE), a 130 kDa band was observed (Figure 5a, Figure S1); this finding indicated that AK is a heterotetramer. Purified AK was subjected to sodium dodecyl sulfate (SDS)-PAGE, and two bands were observed throughout the purification progress (Figure 5b, Figure S2). The 47 and 18 kDa bands corresponded to the AK α- and β-subunits, respectively, which is consistent with the experimental results reported by Kalinowsk *et al.* [18].

### 2.4. Kinetic Assay of the Wild Type (WT) and AK Mutants

As shown in Table 1, kinetic parameters, namely, *V*_max_, *K*_m_, and nH (Hill coefficient), with the substrate l-aspartate, were 96.07 U/mg·s^−1^, 4.56 mol/L, and 2.82, respectively. Based on the dynamic data fitting results, the fitting curve did not conform to the Michaelis–Menten equation but was consistent with the Hill equation. This finding indicated that AK is an allosteric enzyme with positive cooperativity. At the site of the R169 mutant, the *V*_max_ values were 4.71, 2.25, 2.24, and 2.12 times higher than those of the wild type (WT) for R169Y, R169P, R169D, and R169H, respectively. However, positive cooperativity decreased in all mutants. The nH values of R169Y, R169P, R169D, and R169H were 1.13, 1.95, 0.91, and 1.09, respectively, which are consistent with Michaelis–Menten enzymes. The *K*_m_ of each mutant increased relative to WT, which indicated the weakened affinity to l-aspartate. Of the four mutants, the R169Y mutant with the highest activity was selected for further study.

**Table 1:** Kinetic parameters of wild type (WT) and mutants.

Parameters	WT	R169Y	R169P	R169D	R169H
*V*_max_ (U/mg)	96.07	452.78	216.55	208.77	204.25
*K*_m_ (mol·L^−1^)	4.56	4.98	4.95	6.29	8.79
nH	2.82	1.13	1.95	0.91	1.09

### 2.5. Optimum Temperature, pH, and Thermostability of Wild Type (WT) and R169Y

**Figure 6:**
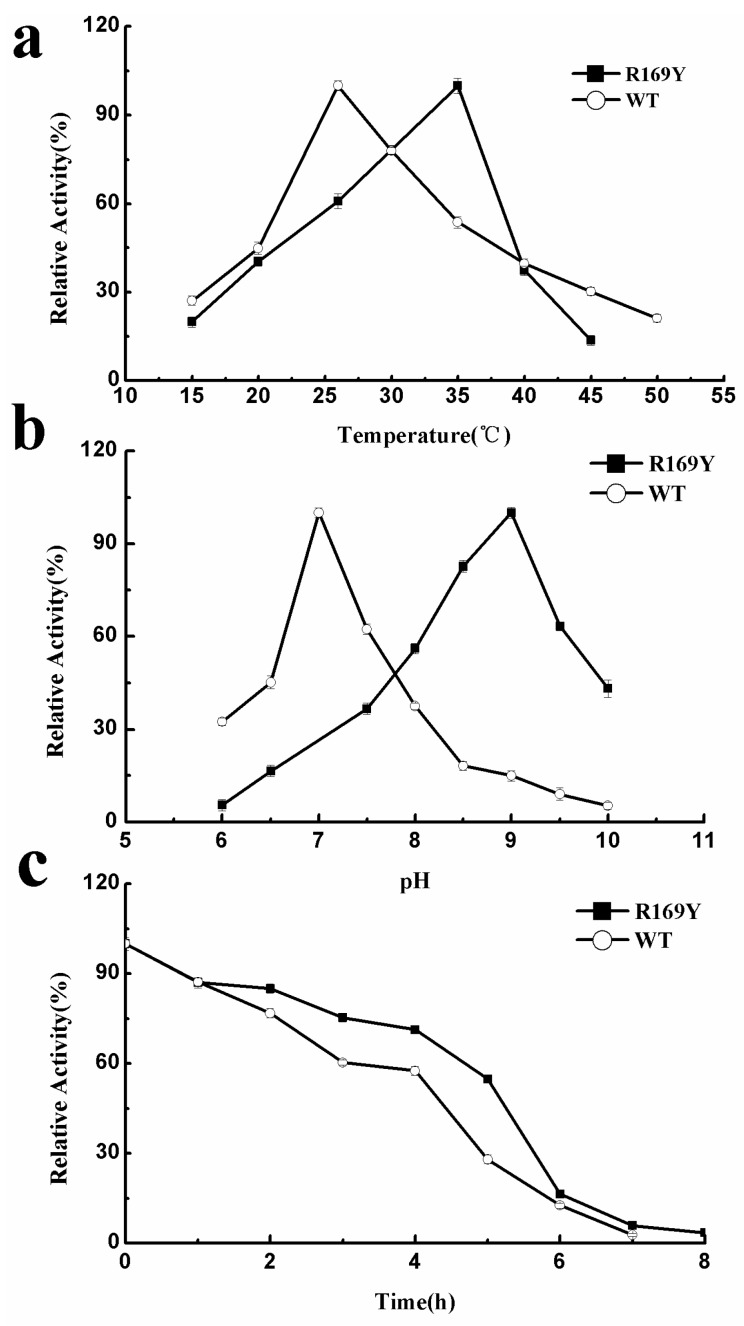
Optimum temperature (**a**); pH (**b**); and thermostability (**c**) curves of WT and R169Y.

l-Aspartate was used as the substrate to study the optimum catalytic conditions of WT and R169Y. As shown in Figure 6a, the optimum reaction temperatures of WT and R169Y were 26 and 35 °C, respectively. The optimum pH values of WT and R169Y were 7.0 and 9.0, respectively. Compared with neutral WT, R169Y became a typical alkaline enzyme when the optimum temperature increased by 9 °C.

The results of thermal stability experiments performed at 26 °C and pH 8.0 are shown in Figure 6c. The half-life of WT was 4.5 h, and the half-life of the mutant R16 9Y increased to 5.5 h. This result indicated that the 3D structure of R169Y is more stable than that of WT.

### 2.6. Effect of Metal Ions and Organic Solvents on WT and R169Y

The effect of metal ions on the activity of WT and the R169Y mutant is shown in Table 2. The concentrations of metal ions investigated were 0.2, 1, 5, and 10 mM. For WT, different concentrations of Ni^2+^, especially 0.2 mM, induced the activation. At this concentration, enzymatic activity increased nearly 1.6-fold. K^+^ improved the activity only at low concentrations (0.2 mM). Other metal ions, including Ca^2+^, Zn^2+^, and Mn^2+^, all exerted inhibitory effects. For the R169Y mutant, enzymatic activity decreased in most of metal ions of various concentrations, except for K^+^, Zn^2+^, Cu^2+^, and Ni^2+^, which promoted the activation of enzymatic activity at 5 mM. Na^+^ and K^+^ slightly increased activity at 1 mM. In summary, the inhibition of the R169Y mutant decreased relative to WT.

The effects of organic solvents on the activity of WT and R169Y are shown in Table 3. The concentrations of organic solvents investigated were 1%, 5%, 10%, and 20%. For WT, concentrations of nearly all organic solvents severely inhibited the enzyme, with inhibition by glycerol being the most significant. Only ethanol significantly activated the enzyme at concentrations of 1%, 5%, and 10%. However, all organic solvents inhibited the enzymatic activity of R169Y.

**Table 2:** Effects of metal ions on WT and R169Y.

Enzyme	WT				R169Y			
	Relative Activity (%)				Relative Activity (%)			
Metal ions (mM)	0.2	1	5	10	0.2	1	5	10
Control	100	100	100	100	100	100	100	100
Na^+^	ND	75.44 ± 0.47	11.40 ± 1.44	5.26 ± 0.33	46.11 ± 0.88	101.48 ± 2.23	89.20 ± 0.43	39.75 ± 0.74
K^+^	137.72 ± 1.54	62.28 ± 1.03	55.26 ± 1.31	45.61 ± 1.04	54.51 ± 1.09	118.10 ± 1.94	105.61 ± 0.98	84.03 ± 1.43
Ca^2+^	57.02 ± 1.09	38.60 ± 0.89	14.91 ± 0.33	10.53 ± 0.43	33.40 ± 0.95	41.60 ± 0.74	92.33 ± 1.45	66.24 ± 1.47
Mg^2+^	54.39 ± 0.87	55.26 ± 1.53	57.90 ± 1.77	43.42 ± 0.74	35.34 ± 1.54	40.74 ± 2.03	76.12 ± 1.78	62.84 ± 1.45
Zn^2+^	90.35 ± 1.89	82.46 ± 1.34	78.07 ± 1.64	71.93 ± 0.45	49.39 ± 0.43	75.54 ± 1.54	164.02 ± 1.95	95.87 ± 0.74
Mn^2+^	78.07 ± 1.54	70.18 ± 2.04	40.35 ± 0.54	10.53 ± 0.32	64.27 ± 1.54	36.71 ± 0.87	18.14 ± 0.32	ND
Cu^2+^	57.02 ± 0.43	42.11 ± 1.54	40.64 ± 0.68	32.46 ± 0.39	24.74 ± 1.43	93.12 ± 1.87	106.84 ± 0.45	73.51 ± 1.54
Fe^3+^	78.07 ± 0.58	44.74 ± 1.54	33.33 ± 1.81	16.67 ± 0.85	86.10 ± 1.73	68.01 ± 0.39	55.73 ± 1.82	ND
Ni^2+^	159.94 ± 1.54	143.57 ± 1.05	129.30 ± 1.74	122.22 ± 2.57	21.34 ± 0.38	75.66 ± 1.47	164.90 ± 0.78	62.08 ± 1.94

ND, enzyme activity could not be determined.

**Table 3:** Effects of organic solvents on WT and R169Y.

Enzyme	WT				R169Y			
	Relative Activity (%)				Relative Activity (%)			
	Concentration (%)				Concentration (%)			
Organic solvents	1	5	10	20	1	5	10	20
Control	100	100	100	100	100	100	100	100
methanol	45.56 ± 1.74	64.44 ± 0.64	113.33 ± 0.54	46.67 ± 0.92	43.04 ± 0.29	5.0 ± 0.32	ND	ND
ethanol	143.33 ± 0.43	182.22 ± 1.54	154.44 ± 2.01	22.22 ± 0.41	30.91 ± 1.94	26.67 ± 1.02	18.84 ± 0.32	7.71 ± 0.55
isopropyl alcohol	30.0 ± 1.56	46.67 ± 2.04	74.45 ± 1.09	67.78 ± 1.87	39.41 ± 0.54	14.07 ± 0.25	ND	ND
*n*-butanol	23.33 ± 0.31	20.0 ± 0.51	ND	ND	30.95 ± 0.93	ND	ND	ND
acetonitrile	41.11 ± 0.43	11.11 ± 0.23	13.33 ± 0.96	ND	42.39 ± 2.06	36.33 ± 1.03	29.48 ± 0.62	16.96 ± 1.09
dimethyl sulfoxide	62.22 ± 1.73	31.11 ± 1.47	13.33 ± 0.95	ND	26.24 ± 1.53	18.46 ± 0.34	16.15 ± 0.49	11.44 ± 1.55
glycerol	25.56 ± 1.07	ND	ND	ND	66.61 ± 1.33	53.83 ± 0.52	37.01 ± 1.54	3.94 ± 0.21

ND, enzyme activity could not be determined.

### 2.7. Effect of Inhibitors on WT and R169Y

CpAK is an allosteric enzyme inhibited by end-products such as Lys, Met, and Thr. Table 4 shows the effect of inhibitors on enzymatic activity when applied at concentrations ranging from 0.2 to 10 mM. The end-products, namely Lys, Met, and Thr, inhibited the enzymatic activity of WT. Inhibitors arranged in ascending order based on inhibitory strength were Thr, Met, and Lys. The results showed that cooperativity inhibition occurred between Met and Lys. For the other groups, such as Thr + Lys, Thr + Met, and Thr + Lys + Met, inhibition decreased compared with the end-products alone. For the R169Y mutant, the inhibition of end-products evidently decreased; by contrast, Lys and Met improved the enzymatic activity by 1.86- and 1.74-fold, respectively. The inhibitor combination Met + Lys exhibited activated cooperativity at different concentrations. The effect of inhibitors on R169Y was weaker than that on WT.

### 2.8. Enzymology Properties of Arg169

Similar to CgAK, CpAK is an allosteric enzyme with Lys, Met, and Thr inhibition and specifically regulated by Lys or Thr, whereas AK in bacilli is regulated by Lys or Lys and Thr [19].

CpAK was successfully expressed in *E. coli*. This neutral enzyme has an optimum temperature of 26 °C and a half-life of 4.5 h. Both Ni^2+^ and ethanol activated AK, and this activation could be used to guide the industrial application of the enzyme. Arg169 influenced enzyme catalysis, which regulates the catalytic domain through a hydrogen bond and therefore affects enzymatic activity. Increased enzyme activity was observed in the R169Y, R169P, R169D, and R169H mutants (Table 1) because resistance from the hydrogen bond between Glu92 and the mutants disappeared; hence, Arg169 participated in nucleophilic catalysis.

Table 4 shows that that AK activity gradually decreased with increasing inhibitor concentration; hence, Lys was found to be the strongest inhibitor. The inhibitor binding site of Lys was found in the substrate binding cavity [20]. Other inhibitors were bound to locations other than in the substrate cavity, which allows them to more easily combine with the substrate than Lys. These inhibitors minimally influenced enzymatic activity mainly because the substrate is not involved in the competition for binding. The arrangement of inhibitors in ascending order based on the strength of the inhibitory effect is as follows: Thr, Met, and Lys. A two-step concerted inhibition of AK by Thr and Lys is therefore proposed. First, Thr binding triggers the interaction of the β-subunit with the regulatory domain in the α-subunit. Second, Lys binding induces a slight rotation in the regulatory domains in the α-subunit [21]. Therefore, the inhibitory effect of this combination mainly depends on Thr. Similarly, our experimental results indicated the absence of evident inhibition differences among the combinations of Thr + Lys, Thr + Met, and Thr + Lys + Met. However, few reports on the inhibitor Met and Met + Lys demonstrated cooperativity [22]. The inhibitory site of Met is probably identical to that of Lys, resulting in a stronger inhibitory effect of Met + Lys than those of Lys or Met alone.

**Table 4:** Effects of inhibitors on WT and R169Y.

Enzyme	WT				R169Y			
	Relative Activity (%)				Relative Activity (%)			
	Concentration (mM)				Concentration (mM)			
Inhibitors	0.2	1	5	10	0.2	1	5	10
Control	100	100	100	100	100	100	100	100
Thr	73.97 ± 0.46	55.86 ± 1.63	27.07 ± 0.54	18.19 ± 0.59	94.25 ± 1.45	79.20 ± 0.54	50.34 ± 1.04	33.82 ± 0.29
Lys	38.47 ± 0.92	20.36 ± 1.03	19.18 ± 0.41	ND	83.09 ± 1.93	186.05 ± 1.22	68.46 ± 1.29	50.63 ± 0.34
Met	53.36 ± 0.56	50.89 ± 0.43	49.58 ± 0.94	43.62 ± 1.65	99.88 ± 1.98	167.43 ± 1.93	116.36 ± 1.19	68.62 ± 1.01
Thr + Lys	88.30 ± 1.14	33.95 ± 1.45	28.63 ± 0.19	30.18 ± 0.82	84.23 ± 0.17	41.13 ± 0.26	37.76 ± 1.38	27.93 ± 0.42
Thr + Met	73.32 ± 1.04	46.52 ± 1.82	29.38 ± 1.05	15.05 ± 0.04	73.04 ± 0.32	48.50 ± 0.91	32.88 ± 0.96	27.46 ± 1.04
Met + Lys	30.86 ± 1.74	22.63 ± 0.43	ND	ND	102.38 ± 2.03	138.47 ± 1.92	146.76 ± 1.03	183.12 ± 1.13
Thr + Lys + Met	93.96 ± 1.54	64.35 ± 1.83	49.04 ± 1.62	35.92 ± 0.81	192.5 ± 2.04	141.99 ± 0.63	30.68 ± 0.14	16.87 ± 0.26

ND, enzyme activity could not be determined.

### 2.9. “Triple Function” of Arg169

**Figure 7:**
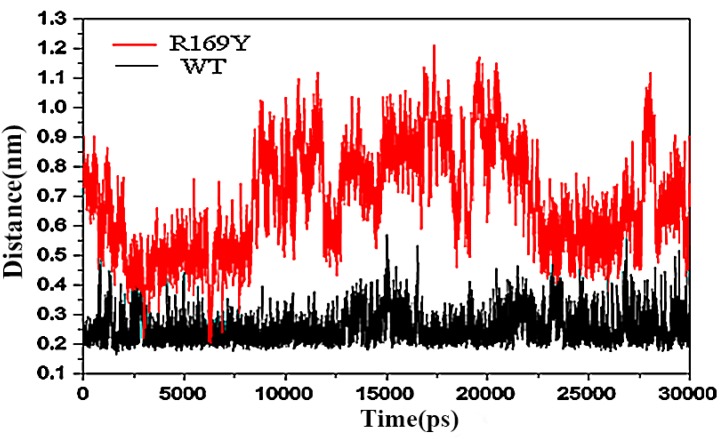
Distance in wild type and R169Y calculated by PyMOL (http://pymol.sourceforge.net/).

The so-called triple function of Arg169 depends on enzyme catalysis with a hydrogen bond (Glu92–Arg169). R169 formed a salt bridge with the catalytic residue Glu 92 in the inactive form. As shown in Figure 7, Glu92 formed a salt bridge with Lys169 in the inactive form (3.0 Å), but no salt bridge was found in the active form (8.6 Å). However, in the mutant R169Y (Figure 7), the salt bridge in the active form was 11.86 Å. This structural difference may lead to differences in the catalytic efficiency of AK.

**Figure 8:**
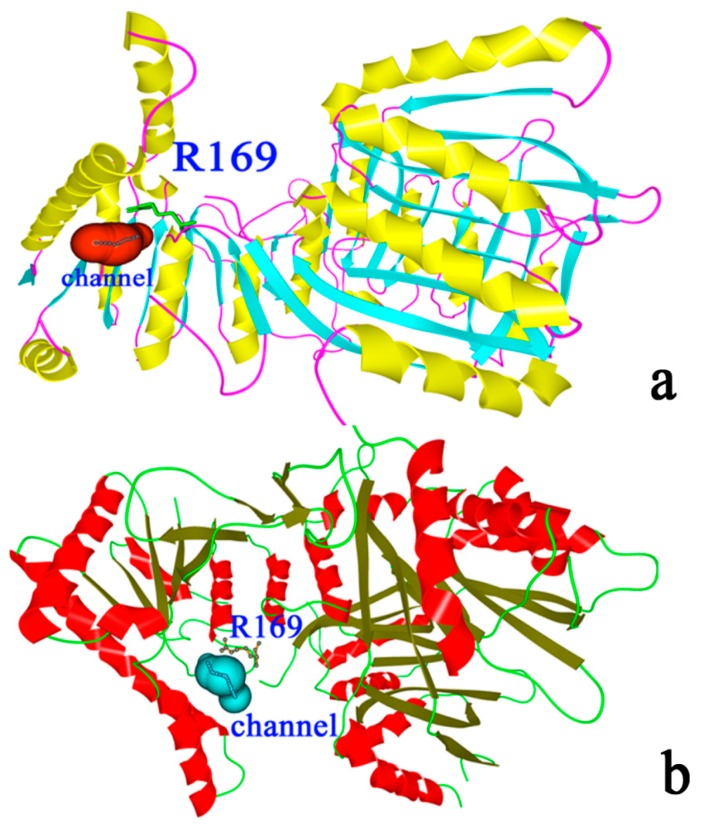
Substrate binding channel of AK. (**a**) Substrate binding in the inactive form of AK, color cyan represents for β-sheet, color yellow represents for α-helix, and color red represents for the channel; (**b**) substrate binding in the active form of AK, color green represents for β-sheet, color red represents for α-helix, and color cyan represents for the channel.

As shown in Figure 8a,b, R169 was located at the substrate channel in the active and inactive forms; hence, this residue can prevent the substrate from sliding. In particular, R169 was absolutely conserved among all AK family members (Figure 1).

CgAK is an allosteric enzyme that shares 98.5% homology with CpAK and exists in two states, namely, relaxed (R) and tense (T) states. In the T state, R151 (α) forms bidentate ionic bonds with E74 (α), and this binding is responsible for aspartate binding. The inhibitor binding structure showed that E3-β1 (β), E74 (α), S41 (α), R151-β5 (α), G152-β5 (α), and S154-β5 (α) were involved in Lys binding [1,2,3,4], and bidentate ionic bonds were formed between R151 and E74. According to homology modeling with CgAK (3aaw) as the template, the R151 (α) and E74 (α) of CgAK correspond to the R169 (α) and E92 (α) of CpAK. For the mutant R169Y, the inhibitory effect of Lys nearly vanished, and this observation may be related to the competition of aspartate and Lys binding. Overall, the binding site of the substrate is similar to that of Lys. Therefore, Arg169 may function as an evolutionary trace.

## 3. Experimental Section

### 3.1. Reagents, Strains, and Plasmids

All restriction enzymes were bought from Promega (Madison, WI, USA). The His-Tag™ monoclonal antibody and the rabbit anti-mouse peroxidase-conjugated secondary antibody were purchased from Merck (San Diego, CA, USA). *E. coli* was obtained from Novagen (Madison, WI, USA). The recombinant plasmid pET-28a-AK was provided by our laboratory.

### 3.2. Construction of Mutant Strains

The genomic DNA of *C. pekinense* was isolated with a genomic DNA extraction kit. The aspartokinase gene was then amplified by PCR, ligated to plasmid PMD 18-T, and then transformed to *E. coli* DH5α. The plasmids were extracted and sequenced. After digestion with the restriction enzymes, namely, BamHI and *Nde*I, the single band between 1000 and 2000 bp was recycled; this band corresponds to the target gene size of 1453 bp. The recombinant plasmid pET-28a-AK was constructed by subcloning the structure gene to pET-28a with His-Tag. Based on the DNA sequence of AK, two primers, namely, 5′-CAGTGGTGTCAGAACCACCNNNACCCAACGTG-3′ and 5′-GTCACCACGTTGGGTNNNGGTGGTTCTGACAC-3′, were designed and synthesized to amplify the gene encoding the mutant R169 site through PCR. The recombinant wild-type plasmid pET-28a-AK was used as template. The following thermal profile was used in PCR: initial denaturation at 94 °C for 4 min, followed by 18 cycles of denaturation at 94 °C for 1 min, annealing at 56 °C for 1 min, and extension at 72 °C for 10 min, and then a final extension of 72 °C for 20 min. The PCR products were digested with *Dpn*I, transformed into *E. coli*, and then cultured in Luria–Bertani (LB) solid medium. The monoclones were inoculated into 96-well plates with 200 µL of the LB medium per well and then transformed into new 96-well plates at a ratio of 2% after overnight culture (37 °C, 180 rpm). The mixture was induced by adding 1 mM isopropyl β-d-1-thiogalactopyranoside (IPTG) at 30 °C for 8 h. The cells were isolated through centrifugation at 3500 rpm for 45 min. The intracellular products were released through multigelation in 50 mM phosphate-buffered saline (PBS, pH 7.4) and then centrifuged at 3500 rpm for 45 min. The supernatant contained the crude enzyme. Enzymatic activity was detected using a microplate reader at 540 nm, and mutations with high activity were screened for sequencing.

### 3.3. Expression and Purification of the Recombinant AK and Mutants

WT and mutant strains were cultured in the LB medium until an absorbance of 0.5 to 0.8 at 600 nm [23,24]. The strains were induced with 1 mM IPTG at 30 °C for 8 h. The cells were isolated through centrifugation at 8000 rpm for 10 min and then suspended in PBS (pH 7.4). The suspension was disrupted through sonication, followed by centrifugation at 8000 rpm for 10 min. The supernatant was filtered using a 0.45 µm membrane to remove residual proteins, loaded onto a Ni^2+^-NTA column, and then eluted with imidazole gradient. The purified enzyme was obtained with 500 mM imidazole. The purity of the WT and mutant samples was analyzed with SDS-PAGE.

### 3.4. Analysis of Native PAGE, SDS-PAGE, and Western Blot

Native PAGE was performed using a polyacrylamide gel consisting of 6% (*w*/*v*) resolving gel and 5% (*w*/*v*) stacking gel. Staining with silver and Coomassie blue were performed to obtain better staining results. SDS-PAGE was performed according to the Laemmli method. In brief, a polyacrylamide gel consisting of 10% (*w*/*v*) resolving gel and 5% (*w*/*v*) stacking gel was used. The gel was then stained with Coomassie Brilliant Blue for visualizing the proteins in the gel [25]. For Western blot analysis, the proteins in the 10% (*w*/*v*) resolving gel were transferred onto a polyvinylidene fluoride membrane through a wet trans-blot system (Bio-Rad, Munich, Germany). The membrane was blocked with 2% nonfat dry milk for 2 h and then incubated for 2 h with HRP Mouse Anti-6X His in TBS at a dilution of 1:2500. The blot was developed with 3,3′-diaminobenzidine tetrahydrochloride.

### 3.5. Steady-State Kinetics Assay of WT and Mutant AKs

The kinetic parameters of WT and mutant AKs were determined through the generation of aspartyl hydroxamate. Each assay tube contained 10 mM l-aspartate, 10.4 mM ATP, 10 mM β-mercaptoethanol, 100 mM Tris-HCl buffer solution (pH 8.0), 1.6 mM MgSO_4_, 800 mM NH_4_OH, 800 mM KCl, and 0.1 mg/mL purified enzyme sample. The assay tubes were incubated at 299 K in a thermocycler for 30 min [26]. The reaction was terminated using isopycnic FeCl_3_. Absorbance was determined at 540 nm. The maximum velocities were measured with l-aspartate concentrations ranging from 1 to 10 mM. The content of AK in the purified enzyme for enzymatic determination was measured using the Bradford method [27]. *K*_m_ value and maximum velocity were determined by fitting the Hill Equation (1) to data points with nonlinear least-squares method. All measurements were performed in triplicate.

(1) $$\text{V}=\frac{{\text{V}}_{\text{max}}\left[\text{S}\right]}{{\text{K}}^{\prime }+{\left[\text{S}\right]}^{\text{n}}}$$

### 3.6. Characterization of AK from WT and Mutant R169Y

The optimum temperature was measured by incubating the reaction mixtures from 15 to 50 °C at 5 °C intervals. The optimum pH was determined using 100 mM Tris-HCl under a wide-range pH buffer of 6 to 10 with a pH 0.5 scale interval [28]. All measurements were performed in triplicate. Standard deviation was used for determination of enzymatic activity.

The effect of metal ions on AK activity was determined by adding reagents containing Na^+^, K^+^, Ca^2+^, Mg^2+^, Zn^2+^, Mn^2+^, Cu^2+^, Fe^3+^, and Ni^2+^ [29]. The ions were analyzed at final concentrations of 0.2, 1, 5, and 10 mM. Enzymatic activity without added metal ions was defined as 100%. All measurements were performed in triplicate.

The effect of organic solvents on AK activity was determined by adding methanol, ethanol, isopropyl alcohol, *n*-butanol, acetonitrile, dimethyl sulfoxide, and glycerol to the enzyme [30]. These organic solvents were investigated at final volume ratios of 1%, 5%, 10%, and 20%. Enzymatic activity without the addition of organic solvents was defined as 100%. All measurements were performed in triplicate.

The effect of inhibitors on AK activity was determined by adding Thr, Lys, Met, Thr + Lys, Thr + Met, Lys + Met, and Thr + Lys + Met to the enzyme. These inhibitors were analyzed at final concentrations of 0.2, 1, 5, and 10 mM. The enzymatic activity without inhibitors was defined as 100%. All measurements were performed in triplicate.

### 3.7. Homology Modeling and Structural Analysis

The sequence of AK was obtained from UniProtKB/Swiss-Prot. AK from *C. glutamicum* (PDB ID 3aaw sequence identity, 99%) was used as the template protein. The BLAST was used for searching, and Swiss Model was used to build the 3D structure [31,32,33]. The distance between the residue of 169 and E92 was calculated with the program PyMOL (http://pymol.sourceforge.net/) for further structural analysis of WT and mutant proteins.

### 3.8. Molecular Docking

The substrate and ATP were docked to the homology modeled AK [10] by using the Lamarckian Genetic Algorithm provided by AutoDock 4.2 software [28,34]. A cubic box was built around the protein with 36 Å × 36 Å × 36 Å points.

### 3.9. Molecular Dynamics (MD) Simulation and Molecular Mechanics-Poisson-Boltzmann Surface Area (MM-PBSA) Calculations

Eleven 10 ns structures of the complex were used as starting points for calculations of binding free energy. All simulations were performed using the Amber 11 package for 10 ns, with the amber 99 sb as the field-force parameter [25]. Binding free energies were calculated using the MM-PBSA method [35]. In addition, the two substrates used in the present study are highly similar. According to previous studies [36,37], the entropy differences should be minimal such that the correlation between the experimental value and the calculated binding free energy may not be substantially improved. Therefore, the solute entropy term was neglected in the present study.

For each MD-simulated complex, we calculated the Δ*G*_bind_ values for 1000 snapshots of the MD simulation trajectory. The final Δ*G*_bind_ value was the average of the Δ*G*_bind_ values for the snapshot trajectory.

## 4. Conclusions

Recombinant AK from *C. pekinense* is a member of the AK superfamily. Experimental data showed that the optimum temperature and pH of AK were 26 °C and pH 7, respectively. The half-life was 4.5 h under the optimum conditions, and ethanol and Ni^2+^ strongly increased the enzymatic activity of CpAK. The steady-state kinetics study confirmed that AK is an allosteric enzyme, and enzymatic activity was inhibited by allosteric inhibitors, such as Lys, Met, and Thr. The results of molecular mechanics-Poisson-Boltzmann surface area (MM-PBSA) showed that the residue Arg169 participated in substrate binding, catalytic domain, and inhibitor binding. These findings can be used to develop new enzymes and provide a basis for amino acid production.

## Supplementary Materials

Supplementary materials can be found at http://www.mdpi.com/1422-0067/16/12/26098/s1.
