# Supplementary material for: Characterization of Aspartate Kinase from Corynebacterium pekinense and the Critical Site of Arg169
Source: Int J Mol Sci. 2015 Nov 27;16(12):28270–84. doi: 10.3390/ijms161226098 (PMC4691045; doi:10.3390/ijms161226098)
Supplement: Supplementary file 1 [file ijms-16-26098-s001.pdf]

Supplementary Materials: Characterization of Aspartate Kinase from *Corynebacterium pekinense* and the Critical Site of Arg169

Weihong Min, Huiying Li, Hongmei Li, Chunlei Liu and Jingsheng Liu

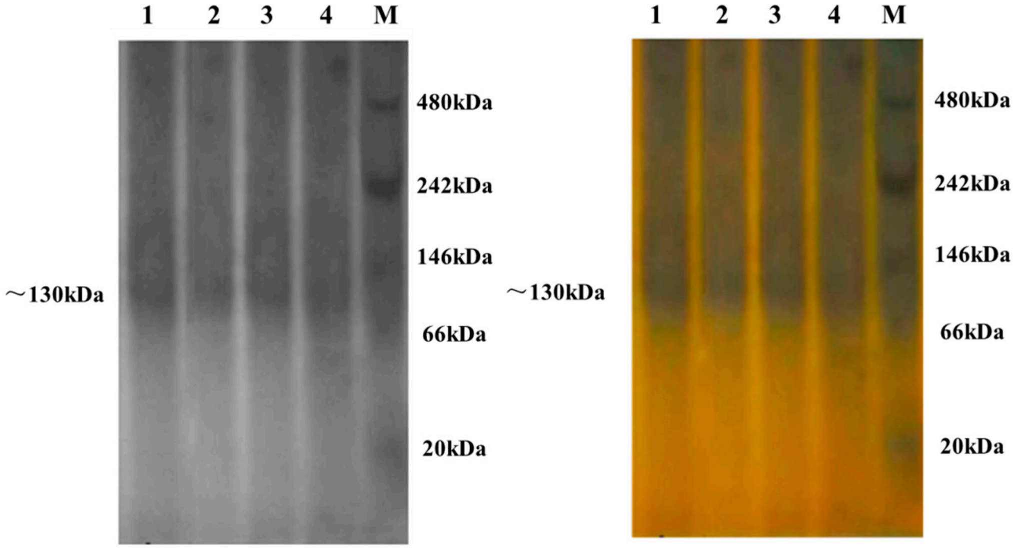

Figure S1. Native polyacrylamide gel electrophoresis of the mutants (silver-stained). M: native marker; lane 1: purified recombinant R169Y; lane 2: purified recombinant R169P; lane 3: purified recombinant R169D; and lane 4: purified recombinant R169H.

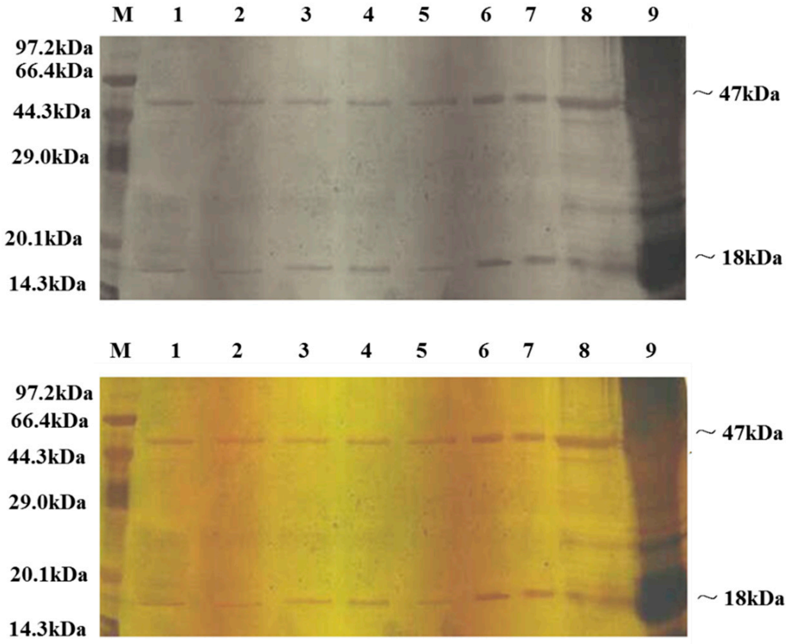

Figure S2. Sodium dodecyl sulfate-polyacrylamide gel electrophoresis of the mutants (silver-staining). M: high-molecular weight protein marker; lanes 1 and 2: purified recombinant R169Y; lanes 3 and 4: purified recombinant R169P; lanes 5 and 6: purified recombinant R169D; lanes 7 and 8: purified recombinant R169H; and lane 9: supernatant of the induced sample.
